# (±)-1-(1-Allyl-1*H*-benzimidazol-2-yl)ethanol

**DOI:** 10.1107/S1600536812044340

**Published:** 2012-11-03

**Authors:** Dong-Ping Li, Min Li, Shuai Li, Hang-Na Hu

**Affiliations:** a Department of Chemistry, Nanchang University, Nanchang 330031, People’s Republic of China

## Abstract

The title compound, C_12_H_14_N_2_O, was obtained by reaction of (±)-1-(1*H*-benzimidazol-2-yl)ethanol with 3-bromo­prop-1-ene. The asymmetric unit contains four crystallographically independent mol­ecules. In the crystal, mol­ecules 1 and 2 are linked *via* O—H⋯N hydrogen bonds, forming chains propagating along [010]. Molecules 3 and 4 are linked to these chains *via* O—H⋯O hydrogen bonds.

## Related literature

For background to the pharmaceutical properties and applications of benzimidazole derivatives, see: Garuti *et al.* (1999 ▶); Matsuno *et al.* (2000 ▶); Stibrany (2001 ▶); Stibrany *et al.* (2002 ▶). For the synthesis of the title compound, see Xia & Xu (2008 ▶).
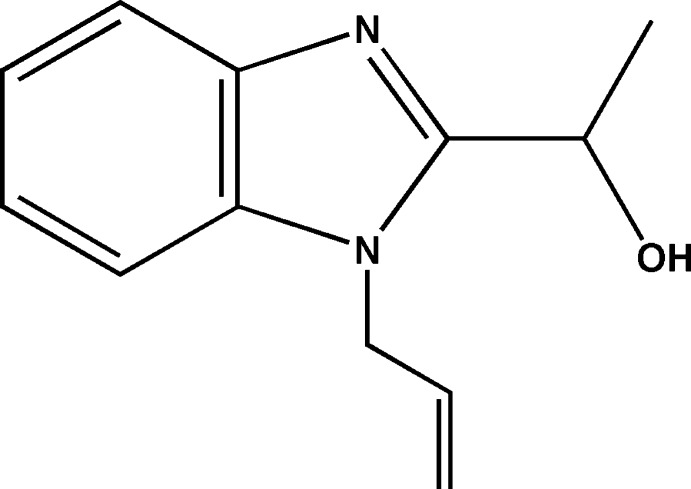


## Experimental

### 

#### Crystal data

C_12_H_14_N_2_O
*M* *_r_* = 202.25
Triclinic, 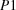

*a* = 8.7816 (18) Å
*b* = 9.1233 (18) Å
*c* = 14.773 (3) Å
α = 96.66 (3)°
β = 107.15 (3)°
γ = 90.83 (3)°
*V* = 1121.8 (4) Å^3^
*Z* = 4
Mo *K*α radiation
μ = 0.08 mm^−1^
*T* = 293 K
0.24 × 0.22 × 0.15 mm

#### Data collection

Rigaku Mercury2 (2x2 bin mode) diffractometer
Absorption correction: multi-scan (*CrystalClear*; Rigaku, 2005 ▶) *T*_min_ = 0.982, *T*_max_ = 0.988
10277 measured reflections
8368 independent reflections
4897 reflections with *I* > 2σ(*I*)
*R*_int_ = 0.031

#### Refinement

*R*[*F* ^2^ > 2σ(*F* ^2^)] = 0.055
*wR*(*F* ^2^) = 0.128
*S* = 1.02
4404 reflections
541 parameters
3 restraints
H-atom parameters constrained
Δρ_max_ = 0.16 e Å^−3^
Δρ_min_ = −0.19 e Å^−3^

### 

Data collection: *CrystalClear* (Rigaku, 2005 ▶); cell refinement: *CrystalClear*; data reduction: *CrystalClear*; program(s) used to solve structure: *SHELXS97* (Sheldrick, 2008 ▶); program(s) used to refine structure: *SHELXL97* (Sheldrick, 2008 ▶); molecular graphics: *SHELXTL* (Sheldrick, 2008 ▶); software used to prepare material for publication: *SHELXL97*.

## Supplementary Material

Crystal structure: contains datablock(s) I, global. DOI: 10.1107/S1600536812044340/rz5017sup1.cif

Structure factors: contains datablock(s) I. DOI: 10.1107/S1600536812044340/rz5017Isup2.hkl

Supplementary material file. DOI: 10.1107/S1600536812044340/rz5017Isup3.cml

Additional supplementary materials:  crystallographic information; 3D view; checkCIF report

## Figures and Tables

**Table 1:** Hydrogen-bond geometry (Å, °)

*D*—H⋯*A*	*D*—H	H⋯*A*	*D*⋯*A*	*D*—H⋯*A*
O1—H1*B*⋯N3^i^	0.82	1.99	2.801 (4)	167
O2—H2*B*⋯N1^ii^	0.82	2.05	2.811 (4)	155
O3—H3*B*⋯O1^iii^	0.82	1.99	2.808 (4)	175
O4—H4*B*⋯O2^i^	0.82	1.97	2.787 (4)	176

Symmetry codes: (i) 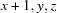; (ii) 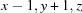; (iii) 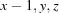.

**Fig. 1.**
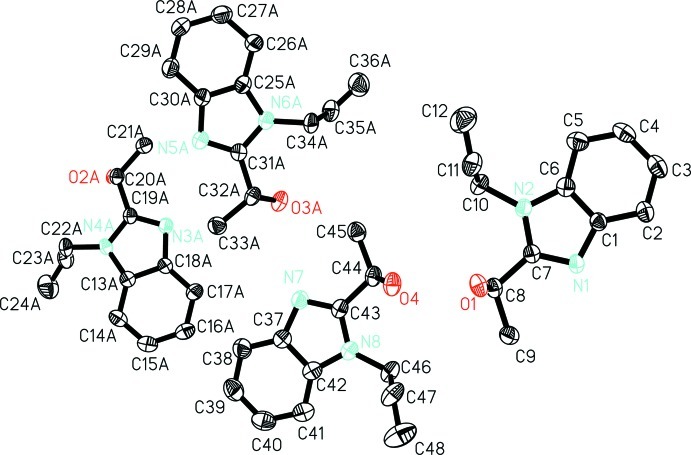
The molecular structure of the title compound with displacement ellipsoids drawn at the 30% probability level. H atoms are omitted for clarity. For clarity purposes, molecules with atoms labelled with suffix A have been generated by the symmetry operation x, y + 1, z.

**Fig. 2.**
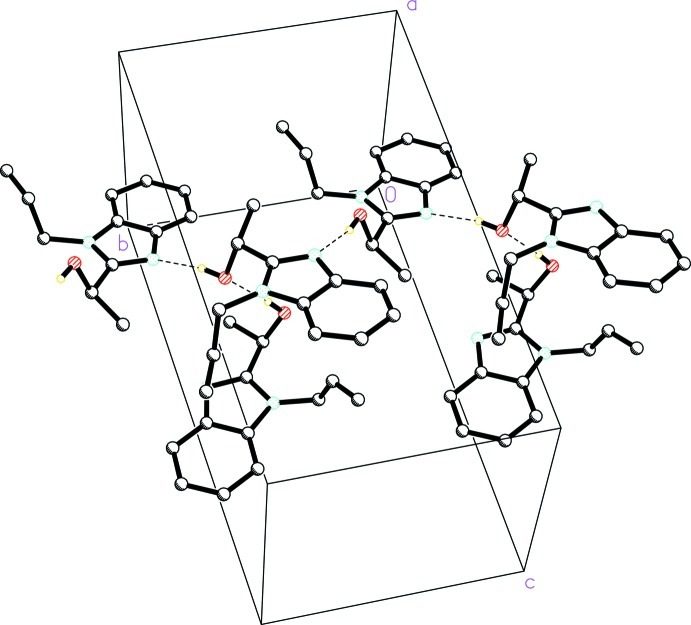
Partial crystal packing of the title compound showing the hydrogen bonding network (dashed lines). H atoms not involved in hydrogen bonding are omitted for clarity.

## supplementary crystallographic information

### Comment

Benzimidazole and its derivatives are important pharmaceutical intermediates and have attracted considerable attention because of good biological and pharmaceutical activities (Matsuno,*et al.*, 2000; Garuti,*et al.*, 1999). These compounds also display other interesting properties as catalysts (Stibrany, 2001) and proton sponges (Stibrany *et al.*, 2002). In this paper, the synthesis and crystal structure of the title compound is reported.

Fig. 1 shows an *ORTEP* plot of the title compound. There are four crystallographically independent molecules in the asymmetric unit. The C═C bonds are within normal ranges. The four benzimidazole ring systems are substantially planar with the the largest deviations from the mean plane of 0.018 (5), 0.008 (5), 0.018 (5) and 0.017 (5) Å for C1–C7/N1/N2, C13–C19/N3/N4, C25–C31/N5/N6 and C37–C43/N7/N8, respectively. In the structure of the title compound (Fig. 2), molecules are connected through intermolecular O—H···N and O—H···O hydrogen bonds into layers parallel to the (110) plane.

### Experimental

All chemicals were obtained from commercial sources and used directly without further purification. (+/-)-1-(1*H*-benzimidazol-2-yl)ethanol was synthesized according to the literature methos (Xia & Xu, 2008). (+/-)-1-(1*H*-benzimidazol-2-yl)ethanol (0.81 g, 5 mmol) was dissolved in acetone (10 ml), then 3-bromoprop-1-ene (0.6 g, 5 mmol) and K_2_CO_3_ (0.83 g, 6 mmol) was added to the solution. The mixture was heated to reflux for 15 h at 70°C. The organic solvent was removed under reduced pressure, and 50 ml chloroform was added. The solid was removed by filtration and washed with chloroform. The combined organic solution was washed with water, dried over anhydrous Na_2_SO_4_, and the solvent removed under reduced pressure. The residues was purified by choromatography column with CHCl_3_ to afford the pure light yellow solid (0.745 g, yield: 74%). ^1^H-NMR(CDCl_3_, 500 MHz): δ1.69 (d, 3 H), 3.66(br, 1H), 4.80(m, 2H), 4.98(d, 1H), 5.11 (q, 1 H), 5.21(d, 1 H), 5.94(m, 1 H), 7.25(m, 3H), 7.73 (m, 1 H). Single crystals of the title compound suitable for X-ray diffraction analysis were obtained from a methanol solution by slow evaporation after a week.

### Refinement

All H atoms attached to C and O atom were fixed geometrically and treated as riding, with C—H = 0.98 Å (methine), 0.96 Å (methyl) or 0.93 Å(aromatic), O—H = 0.82 Å, and with *U*_iso_ (H) = 1.2*U*_eq_ (C) or 1.5*U*_eq_(C, O) for methyl and hydroxy H atoms. In the absence of significant anomalous scattering, 3937 Friedel pairs have been merged.

### Crystal data

**Table:**

C_12_H_14_N_2_O	*V* = 1121.8 (4) Å^3^
*M**_r_* = 202.25	*Z* = 4
Triclinic, *P*1	*F*(000) = 432
Hall symbol: P 1	*D*_x_ = 1.198 Mg m^−^^3^
*a* = 8.7816 (18) Å	Mo *K*α radiation, λ = 0.71073 Å
*b* = 9.1233 (18) Å	θ = 2.4–28.0°
*c* = 14.773 (3) Å	µ = 0.08 mm^−^^1^
α = 96.66 (3)°	*T* = 293 K
β = 107.15 (3)°	Block, colourless
γ = 90.83 (3)°	0.24 × 0.22 × 0.15 mm

### Data collection

**Table:**

Rigaku Mercury2 (2x2 bin mode) diffractometer	8368 independent reflections
Radiation source: fine-focus sealed tube	4897 reflections with *I* > 2σ(*I*)
Graphite monochromator	*R*_int_ = 0.031
Detector resolution: 13.6612 pixels mm^-1^	θ_max_ = 26.0°, θ_min_ = 2.4°
ω scans	*h* = −10→10
Absorption correction: multi-scan (*CrystalClear*; Rigaku, 2005)	*k* = −11→11
*T*_min_ = 0.982, *T*_max_ = 0.988	*l* = −18→18
10277 measured reflections	

### Refinement

**Table:**

Refinement on *F*^2^	Primary atom site location: structure-invariant direct methods
Least-squares matrix: full	Secondary atom site location: difference Fourier map
*R*[*F*^2^ > 2σ(*F*^2^)] = 0.055	Hydrogen site location: inferred from neighbouring sites
*wR*(*F*^2^) = 0.128	H-atom parameters constrained
*S* = 1.02	*w* = 1/[σ^2^(*F*_o_^2^) + (0.0586*P*)^2^] where *P* = (*F*_o_^2^ + 2*F*_c_^2^)/3
4404 reflections	(Δ/σ)_max_ < 0.001
541 parameters	Δρ_max_ = 0.16 e Å^−^^3^
3 restraints	Δρ_min_ = −0.19 e Å^−^^3^

### Special details

**Table:**

Geometry. All e.s.d.'s (except the e.s.d. in the dihedral angle between two l.s. planes) are estimated using the full covariance matrix. The cell e.s.d.'s are taken into account individually in the estimation of e.s.d.'s in distances, angles and torsion angles; correlations between e.s.d.'s in cell parameters are only used when they are defined by crystal symmetry. An approximate (isotropic) treatment of cell e.s.d.'s is used for estimating e.s.d.'s involving l.s. planes.
Refinement. Refinement of *F*^2^ against ALL reflections. The weighted *R*-factor *wR* and goodness of fit *S* are based on *F*^2^, conventional *R*-factors *R* are based on *F*, with *F* set to zero for negative *F*^2^. The threshold expression of *F*^2^ > σ(*F*^2^) is used only for calculating *R*-factors(gt) *etc*. and is not relevant to the choice of reflections for refinement. *R*-factors based on *F*^2^ are statistically about twice as large as those based on *F*, and *R*- factors based on ALL data will be even larger.

### Fractional atomic coordinates and isotropic or equivalent isotropic displacement parameters (Å^2^)

**Table:**

	*x*	*y*	*z*	*U*_iso_*/*U*_eq_	
C1	0.5685 (5)	0.0013 (4)	0.2505 (3)	0.0458 (10)	
C2	0.5232 (6)	−0.1444 (5)	0.2097 (3)	0.0582 (12)	
H2A	0.5454	−0.2222	0.2463	0.070*	
C3	0.4441 (6)	−0.1693 (6)	0.1128 (4)	0.0690 (14)	
H3A	0.4135	−0.2654	0.0835	0.083*	
C4	0.4100 (6)	−0.0526 (6)	0.0590 (3)	0.0712 (14)	
H4A	0.3558	−0.0731	−0.0058	0.085*	
C5	0.4531 (6)	0.0936 (6)	0.0974 (3)	0.0643 (13)	
H5A	0.4310	0.1708	0.0603	0.077*	
C6	0.5318 (5)	0.1171 (5)	0.1951 (3)	0.0489 (10)	
C7	0.6574 (4)	0.2036 (4)	0.3452 (3)	0.0435 (10)	
C8	0.7359 (5)	0.3134 (4)	0.4309 (3)	0.0479 (10)	
H8A	0.6697	0.3990	0.4307	0.058*	
C9	0.7615 (6)	0.2491 (5)	0.5242 (3)	0.0594 (12)	
H9A	0.8118	0.3233	0.5767	0.089*	
H9B	0.8284	0.1667	0.5254	0.089*	
H9C	0.6604	0.2167	0.5296	0.089*	
C10	0.5846 (5)	0.3949 (5)	0.2284 (3)	0.0556 (12)	
H10A	0.6017	0.4673	0.2844	0.067*	
H10B	0.4805	0.4084	0.1848	0.067*	
C11	0.7095 (7)	0.4188 (5)	0.1814 (4)	0.0688 (13)	
H11A	0.8146	0.4042	0.2150	0.083*	
C12	0.6823 (9)	0.4589 (7)	0.0964 (5)	0.104 (2)	
H12A	0.5786	0.4745	0.0607	0.125*	
H12B	0.7665	0.4719	0.0715	0.125*	
C13	0.1628 (5)	0.7216 (5)	0.6969 (3)	0.0472 (10)	
C14	0.2453 (5)	0.7395 (6)	0.7940 (3)	0.0627 (13)	
H14A	0.2719	0.8327	0.8289	0.075*	
C15	0.2858 (6)	0.6124 (6)	0.8360 (3)	0.0683 (14)	
H15A	0.3421	0.6202	0.9006	0.082*	
C16	0.2449 (6)	0.4733 (6)	0.7843 (4)	0.0662 (13)	
H16A	0.2742	0.3900	0.8150	0.079*	
C17	0.1610 (5)	0.4556 (5)	0.6878 (3)	0.0560 (12)	
H17A	0.1338	0.3620	0.6534	0.067*	
C18	0.1189 (5)	0.5824 (4)	0.6439 (3)	0.0445 (10)	
C19	0.0315 (4)	0.7439 (4)	0.5469 (3)	0.0428 (10)	
C20	−0.0486 (5)	0.8173 (4)	0.4616 (3)	0.0478 (10)	
H20A	0.0186	0.9028	0.4596	0.057*	
C21	−0.0795 (6)	0.7139 (5)	0.3688 (3)	0.0610 (12)	
H21A	−0.1311	0.7656	0.3160	0.091*	
H21B	−0.1469	0.6307	0.3699	0.091*	
H21C	0.0201	0.6800	0.3621	0.091*	
C22	0.1121 (5)	0.9846 (4)	0.6599 (4)	0.0564 (12)	
H22A	0.0953	1.0334	0.6030	0.068*	
H22B	0.2169	1.0169	0.7029	0.068*	
C23	−0.0127 (6)	1.0274 (5)	0.7075 (4)	0.0688 (14)	
H23A	−0.1190	1.0031	0.6733	0.083*	
C24	0.0180 (9)	1.0959 (7)	0.7930 (5)	0.106 (2)	
H24A	0.1230	1.1218	0.8290	0.127*	
H24B	−0.0649	1.1194	0.8184	0.127*	
C25	0.1235 (5)	0.2875 (5)	0.1527 (3)	0.0510 (11)	
C26	0.0739 (6)	0.2591 (5)	0.0539 (3)	0.0646 (13)	
H26A	0.0475	0.1639	0.0220	0.077*	
C27	0.0664 (7)	0.3815 (6)	0.0058 (4)	0.0737 (14)	
H27A	0.0336	0.3679	−0.0606	0.088*	
C28	0.1059 (6)	0.5232 (5)	0.0528 (4)	0.0726 (15)	
H28A	0.0992	0.6020	0.0173	0.087*	
C29	0.1547 (6)	0.5505 (5)	0.1507 (4)	0.0672 (13)	
H29A	0.1803	0.6462	0.1820	0.081*	
C30	0.1644 (5)	0.4289 (5)	0.2017 (3)	0.0525 (11)	
C31	0.2025 (5)	0.2829 (4)	0.3089 (3)	0.0466 (10)	
C32	0.2450 (5)	0.2198 (5)	0.4023 (3)	0.0508 (10)	
H32A	0.3255	0.1470	0.4008	0.061*	
C33	0.3171 (6)	0.3359 (5)	0.4866 (3)	0.0616 (12)	
H33A	0.3422	0.2909	0.5445	0.092*	
H33B	0.2421	0.4108	0.4887	0.092*	
H33C	0.4127	0.3798	0.4801	0.092*	
C34	0.1221 (6)	0.0331 (4)	0.2065 (4)	0.0594 (12)	
H34A	0.1757	−0.0082	0.2645	0.071*	
H34B	0.1681	−0.0067	0.1572	0.071*	
C35	−0.0505 (6)	−0.0127 (5)	0.1766 (4)	0.0705 (14)	
H35A	−0.1110	0.0278	0.2145	0.085*	
C36	−0.1226 (8)	−0.1036 (6)	0.1027 (5)	0.0980 (19)	
H36A	−0.0662	−0.1465	0.0630	0.118*	
H36B	−0.2312	−0.1267	0.0889	0.118*	
C37	0.5376 (5)	1.0456 (5)	0.6951 (3)	0.0533 (11)	
C38	0.5520 (6)	1.1886 (6)	0.7412 (4)	0.0732 (15)	
H38A	0.5271	1.2690	0.7072	0.088*	
C39	0.6052 (7)	1.2070 (6)	0.8401 (4)	0.0818 (16)	
H39A	0.6149	1.3016	0.8729	0.098*	
C40	0.6444 (7)	1.0866 (7)	0.8916 (4)	0.0816 (16)	
H40A	0.6803	1.1032	0.9579	0.098*	
C41	0.6314 (6)	0.9450 (6)	0.8468 (4)	0.0728 (14)	
H41A	0.6571	0.8650	0.8812	0.087*	
C42	0.5785 (5)	0.9265 (5)	0.7483 (3)	0.0562 (12)	
C43	0.4930 (5)	0.8496 (5)	0.5936 (3)	0.0512 (11)	
C44	0.4472 (5)	0.7419 (5)	0.5029 (3)	0.0537 (11)	
H44A	0.3670	0.6703	0.5084	0.064*	
C45	0.3721 (6)	0.8175 (5)	0.4162 (3)	0.0629 (12)	
H45A	0.3447	0.7459	0.3602	0.094*	
H45B	0.4463	0.8915	0.4104	0.094*	
H45C	0.2774	0.8631	0.4228	0.094*	
C46	0.5790 (6)	0.6494 (5)	0.7029 (4)	0.0667 (14)	
H46A	0.5206	0.5808	0.6480	0.080*	
H46B	0.5404	0.6320	0.7561	0.080*	
C47	0.7517 (7)	0.6209 (6)	0.7272 (4)	0.0810 (16)	
H47A	0.8037	0.6350	0.6821	0.097*	
C48	0.8330 (9)	0.5788 (9)	0.8051 (5)	0.121 (3)	
H48A	0.7847	0.5637	0.8517	0.146*	
H48B	0.9409	0.5630	0.8157	0.146*	
N1	0.6487 (4)	0.0580 (4)	0.3448 (2)	0.0471 (8)	
N2	0.5884 (4)	0.2455 (3)	0.2568 (2)	0.0468 (8)	
N3	0.0372 (4)	0.5997 (4)	0.5496 (2)	0.0470 (8)	
N4	0.1055 (4)	0.8238 (4)	0.6335 (2)	0.0456 (8)	
N5	0.2142 (4)	0.4240 (4)	0.3004 (3)	0.0535 (9)	
N6	0.1496 (4)	0.1939 (4)	0.2233 (3)	0.0515 (9)	
N7	0.4845 (4)	0.9947 (4)	0.5973 (3)	0.0573 (10)	
N8	0.5482 (4)	0.8010 (4)	0.6817 (3)	0.0547 (9)	
O1	0.8842 (3)	0.3584 (3)	0.4171 (2)	0.0575 (8)	
H1B	0.9230	0.4234	0.4617	0.069*	
O2	−0.1956 (3)	0.8682 (3)	0.4758 (2)	0.0577 (8)	
H2B	−0.2118	0.9311	0.4392	0.069*	
O3	0.1079 (4)	0.1432 (3)	0.4119 (2)	0.0636 (8)	
H3B	0.0389	0.2020	0.4139	0.095*	
O4	0.5825 (4)	0.6616 (3)	0.4948 (2)	0.0656 (9)	
H4B	0.6509	0.7186	0.4889	0.098*	

### Atomic displacement parameters (Å^2^)

**Table:**

	*U*^11^	*U*^22^	*U*^33^	*U*^12^	*U*^13^	*U*^23^
C1	0.044 (2)	0.043 (2)	0.048 (2)	−0.0030 (18)	0.010 (2)	0.0025 (19)
C2	0.063 (3)	0.047 (3)	0.061 (3)	−0.004 (2)	0.017 (3)	−0.003 (2)
C3	0.073 (3)	0.062 (3)	0.067 (3)	−0.014 (3)	0.022 (3)	−0.014 (3)
C4	0.072 (3)	0.084 (4)	0.047 (3)	−0.010 (3)	0.009 (3)	−0.011 (3)
C5	0.062 (3)	0.074 (3)	0.053 (3)	−0.002 (3)	0.011 (2)	0.007 (3)
C6	0.037 (2)	0.052 (3)	0.057 (3)	−0.0002 (19)	0.012 (2)	0.006 (2)
C7	0.036 (2)	0.043 (2)	0.051 (3)	0.0041 (18)	0.013 (2)	0.002 (2)
C8	0.041 (2)	0.043 (2)	0.058 (3)	0.0033 (18)	0.014 (2)	0.0006 (19)
C9	0.069 (3)	0.059 (3)	0.048 (3)	−0.004 (2)	0.016 (2)	0.004 (2)
C10	0.054 (3)	0.047 (3)	0.069 (3)	0.007 (2)	0.017 (2)	0.019 (2)
C11	0.074 (3)	0.054 (3)	0.084 (4)	−0.005 (2)	0.030 (3)	0.016 (3)
C12	0.133 (6)	0.095 (5)	0.102 (5)	0.005 (4)	0.056 (4)	0.032 (4)
C13	0.043 (2)	0.049 (2)	0.051 (3)	0.0064 (19)	0.017 (2)	0.005 (2)
C14	0.056 (3)	0.070 (3)	0.053 (3)	0.002 (3)	0.007 (2)	−0.004 (2)
C15	0.066 (3)	0.086 (4)	0.049 (3)	0.005 (3)	0.012 (3)	0.008 (3)
C16	0.063 (3)	0.075 (3)	0.064 (3)	0.015 (3)	0.016 (3)	0.025 (3)
C17	0.054 (3)	0.055 (3)	0.061 (3)	0.009 (2)	0.017 (2)	0.015 (2)
C18	0.041 (2)	0.045 (2)	0.048 (2)	0.0037 (18)	0.015 (2)	0.0018 (19)
C19	0.033 (2)	0.043 (2)	0.052 (3)	−0.0012 (17)	0.014 (2)	0.0045 (19)
C20	0.048 (2)	0.040 (2)	0.056 (3)	0.0050 (19)	0.016 (2)	0.0061 (19)
C21	0.077 (3)	0.055 (3)	0.052 (3)	0.005 (2)	0.019 (2)	0.009 (2)
C22	0.059 (3)	0.038 (2)	0.069 (3)	−0.001 (2)	0.018 (2)	−0.002 (2)
C23	0.072 (3)	0.052 (3)	0.083 (4)	0.008 (2)	0.030 (3)	−0.007 (3)
C24	0.123 (6)	0.095 (5)	0.106 (5)	0.005 (4)	0.057 (4)	−0.020 (4)
C25	0.049 (2)	0.047 (2)	0.062 (3)	0.011 (2)	0.022 (2)	0.011 (2)
C26	0.081 (4)	0.057 (3)	0.056 (3)	0.009 (3)	0.020 (3)	0.008 (2)
C27	0.085 (4)	0.077 (4)	0.060 (3)	0.013 (3)	0.019 (3)	0.018 (3)
C28	0.089 (4)	0.063 (3)	0.067 (3)	0.003 (3)	0.019 (3)	0.024 (3)
C29	0.075 (3)	0.051 (3)	0.076 (4)	0.001 (2)	0.021 (3)	0.013 (2)
C30	0.051 (3)	0.049 (3)	0.058 (3)	0.001 (2)	0.017 (2)	0.010 (2)
C31	0.041 (2)	0.047 (2)	0.054 (3)	0.0046 (19)	0.016 (2)	0.006 (2)
C32	0.047 (2)	0.044 (2)	0.067 (3)	0.0087 (19)	0.022 (2)	0.016 (2)
C33	0.058 (3)	0.063 (3)	0.058 (3)	0.005 (2)	0.010 (2)	0.006 (2)
C34	0.065 (3)	0.039 (2)	0.074 (3)	0.006 (2)	0.020 (3)	0.005 (2)
C35	0.072 (3)	0.056 (3)	0.089 (4)	−0.005 (3)	0.033 (3)	0.010 (3)
C36	0.085 (4)	0.088 (4)	0.108 (5)	−0.008 (4)	0.010 (4)	0.009 (4)
C37	0.044 (2)	0.058 (3)	0.061 (3)	0.009 (2)	0.019 (2)	0.010 (2)
C38	0.078 (4)	0.059 (3)	0.076 (4)	0.009 (3)	0.015 (3)	0.005 (3)
C39	0.086 (4)	0.072 (4)	0.082 (4)	0.007 (3)	0.026 (3)	−0.011 (3)
C40	0.079 (4)	0.107 (5)	0.060 (3)	0.006 (3)	0.025 (3)	0.007 (3)
C41	0.074 (4)	0.080 (4)	0.068 (3)	0.009 (3)	0.021 (3)	0.019 (3)
C42	0.048 (3)	0.065 (3)	0.061 (3)	0.007 (2)	0.021 (2)	0.017 (2)
C43	0.042 (2)	0.047 (3)	0.067 (3)	0.003 (2)	0.017 (2)	0.013 (2)
C44	0.045 (3)	0.045 (2)	0.073 (3)	0.000 (2)	0.021 (2)	0.008 (2)
C45	0.055 (3)	0.060 (3)	0.069 (3)	0.002 (2)	0.013 (2)	0.002 (2)
C46	0.066 (3)	0.055 (3)	0.085 (4)	0.008 (2)	0.024 (3)	0.028 (3)
C47	0.075 (4)	0.075 (4)	0.107 (4)	0.026 (3)	0.035 (3)	0.043 (3)
C48	0.093 (5)	0.158 (7)	0.129 (6)	0.038 (5)	0.034 (5)	0.076 (5)
N1	0.049 (2)	0.0373 (19)	0.053 (2)	0.0011 (16)	0.0136 (18)	0.0013 (16)
N2	0.045 (2)	0.0405 (19)	0.052 (2)	0.0014 (15)	0.0100 (17)	0.0063 (16)
N3	0.045 (2)	0.043 (2)	0.052 (2)	0.0017 (16)	0.0121 (17)	0.0070 (16)
N4	0.045 (2)	0.0398 (19)	0.050 (2)	0.0014 (15)	0.0148 (17)	−0.0013 (16)
N5	0.056 (2)	0.047 (2)	0.060 (2)	0.0061 (17)	0.0182 (19)	0.0114 (17)
N6	0.052 (2)	0.042 (2)	0.061 (2)	0.0063 (16)	0.0168 (19)	0.0099 (18)
N7	0.060 (2)	0.047 (2)	0.064 (3)	0.0065 (18)	0.016 (2)	0.0099 (18)
N8	0.053 (2)	0.051 (2)	0.065 (3)	0.0076 (18)	0.021 (2)	0.0155 (19)
O1	0.0543 (18)	0.0485 (17)	0.0662 (19)	−0.0111 (14)	0.0192 (15)	−0.0076 (14)
O2	0.0560 (18)	0.0567 (18)	0.0671 (19)	0.0160 (14)	0.0230 (15)	0.0213 (15)
O3	0.0650 (19)	0.0506 (17)	0.083 (2)	0.0016 (15)	0.0296 (17)	0.0195 (16)
O4	0.0586 (19)	0.0491 (17)	0.092 (2)	0.0072 (15)	0.0274 (18)	0.0055 (16)

### Geometric parameters (Å, º)

**Table:**

C1—C6	1.394 (6)	C25—N6	1.393 (5)
C1—C2	1.395 (6)	C26—C27	1.384 (7)
C1—N1	1.396 (5)	C26—H26A	0.9300
C2—C3	1.385 (7)	C27—C28	1.383 (7)
C2—H2A	0.9300	C27—H27A	0.9300
C3—C4	1.385 (7)	C28—C29	1.373 (7)
C3—H3A	0.9300	C28—H28A	0.9300
C4—C5	1.388 (7)	C29—C30	1.402 (6)
C4—H4A	0.9300	C29—H29A	0.9300
C5—C6	1.394 (6)	C30—N5	1.399 (6)
C5—H5A	0.9300	C31—N5	1.314 (5)
C6—N2	1.385 (5)	C31—N6	1.371 (5)
C7—N1	1.329 (5)	C31—C32	1.505 (6)
C7—N2	1.369 (5)	C32—O3	1.434 (5)
C7—C8	1.501 (6)	C32—C33	1.510 (6)
C8—O1	1.438 (5)	C32—H32A	0.9800
C8—C9	1.517 (6)	C33—H33A	0.9600
C8—H8A	0.9800	C33—H33B	0.9600
C9—H9A	0.9600	C33—H33C	0.9600
C9—H9B	0.9600	C34—N6	1.463 (5)
C9—H9C	0.9600	C34—C35	1.486 (7)
C10—N2	1.470 (5)	C34—H34A	0.9700
C10—C11	1.487 (7)	C34—H34B	0.9700
C10—H10A	0.9700	C35—C36	1.284 (7)
C10—H10B	0.9700	C35—H35A	0.9300
C11—C12	1.303 (8)	C36—H36A	0.9300
C11—H11A	0.9300	C36—H36B	0.9300
C12—H12A	0.9300	C37—C38	1.386 (6)
C12—H12B	0.9300	C37—N7	1.400 (6)
C13—N4	1.392 (5)	C37—C42	1.404 (6)
C13—C14	1.392 (6)	C38—C39	1.384 (7)
C13—C18	1.397 (5)	C38—H38A	0.9300
C14—C15	1.380 (7)	C39—C40	1.398 (8)
C14—H14A	0.9300	C39—H39A	0.9300
C15—C16	1.387 (7)	C40—C41	1.368 (8)
C15—H15A	0.9300	C40—H40A	0.9300
C16—C17	1.388 (7)	C41—C42	1.379 (7)
C16—H16A	0.9300	C41—H41A	0.9300
C17—C18	1.393 (6)	C42—N8	1.388 (6)
C17—H17A	0.9300	C43—N7	1.323 (5)
C18—N3	1.395 (5)	C43—N8	1.376 (5)
C19—N3	1.322 (5)	C43—C44	1.512 (6)
C19—N4	1.368 (5)	C44—O4	1.432 (5)
C19—C20	1.489 (6)	C44—C45	1.508 (6)
C20—O2	1.443 (5)	C44—H44A	0.9800
C20—C21	1.522 (6)	C45—H45A	0.9600
C20—H20A	0.9800	C45—H45B	0.9600
C21—H21A	0.9600	C45—H45C	0.9600
C21—H21B	0.9600	C46—N8	1.466 (5)
C21—H21C	0.9600	C46—C47	1.487 (7)
C22—N4	1.469 (5)	C46—H46A	0.9700
C22—C23	1.501 (6)	C46—H46B	0.9700
C22—H22A	0.9700	C47—C48	1.268 (8)
C22—H22B	0.9700	C47—H47A	0.9300
C23—C24	1.292 (7)	C48—H48A	0.9300
C23—H23A	0.9300	C48—H48B	0.9300
C24—H24A	0.9300	O1—H1B	0.8201
C24—H24B	0.9300	O2—H2B	0.8197
C25—C26	1.386 (6)	O3—H3B	0.8200
C25—C30	1.391 (6)	O4—H4B	0.8200
			
C6—C1—C2	120.6 (4)	C29—C28—H28A	119.2
C6—C1—N1	109.5 (3)	C27—C28—H28A	119.2
C2—C1—N1	130.0 (4)	C28—C29—C30	117.5 (4)
C3—C2—C1	117.7 (4)	C28—C29—H29A	121.2
C3—C2—H2A	121.2	C30—C29—H29A	121.2
C1—C2—H2A	121.2	C25—C30—N5	110.6 (4)
C2—C3—C4	120.7 (4)	C25—C30—C29	119.8 (4)
C2—C3—H3A	119.6	N5—C30—C29	129.6 (4)
C4—C3—H3A	119.6	N5—C31—N6	113.8 (4)
C3—C4—C5	123.0 (5)	N5—C31—C32	124.7 (4)
C3—C4—H4A	118.5	N6—C31—C32	121.5 (3)
C5—C4—H4A	118.5	O3—C32—C31	110.0 (3)
C4—C5—C6	115.7 (5)	O3—C32—C33	111.8 (4)
C4—C5—H5A	122.2	C31—C32—C33	112.3 (4)
C6—C5—H5A	122.2	O3—C32—H32A	107.5
N2—C6—C5	131.6 (4)	C31—C32—H32A	107.5
N2—C6—C1	106.1 (4)	C33—C32—H32A	107.5
C5—C6—C1	122.3 (4)	C32—C33—H33A	109.5
N1—C7—N2	112.5 (3)	C32—C33—H33B	109.5
N1—C7—C8	125.2 (4)	H33A—C33—H33B	109.5
N2—C7—C8	122.3 (4)	C32—C33—H33C	109.5
O1—C8—C7	104.7 (3)	H33A—C33—H33C	109.5
O1—C8—C9	111.9 (3)	H33B—C33—H33C	109.5
C7—C8—C9	112.7 (3)	N6—C34—C35	112.3 (4)
O1—C8—H8A	109.2	N6—C34—H34A	109.1
C7—C8—H8A	109.2	C35—C34—H34A	109.1
C9—C8—H8A	109.2	N6—C34—H34B	109.1
C8—C9—H9A	109.5	C35—C34—H34B	109.1
C8—C9—H9B	109.5	H34A—C34—H34B	107.9
H9A—C9—H9B	109.5	C36—C35—C34	125.2 (5)
C8—C9—H9C	109.5	C36—C35—H35A	117.4
H9A—C9—H9C	109.5	C34—C35—H35A	117.4
H9B—C9—H9C	109.5	C35—C36—H36A	120.0
N2—C10—C11	110.9 (4)	C35—C36—H36B	120.0
N2—C10—H10A	109.5	H36A—C36—H36B	120.0
C11—C10—H10A	109.5	C38—C37—N7	129.6 (4)
N2—C10—H10B	109.5	C38—C37—C42	120.1 (5)
C11—C10—H10B	109.5	N7—C37—C42	110.3 (4)
H10A—C10—H10B	108.0	C39—C38—C37	117.4 (5)
C12—C11—C10	124.7 (6)	C39—C38—H38A	121.3
C12—C11—H11A	117.7	C37—C38—H38A	121.3
C10—C11—H11A	117.7	C38—C39—C40	121.5 (5)
C11—C12—H12A	120.0	C38—C39—H39A	119.2
C11—C12—H12B	120.0	C40—C39—H39A	119.2
H12A—C12—H12B	120.0	C41—C40—C39	121.6 (5)
N4—C13—C14	131.7 (4)	C41—C40—H40A	119.2
N4—C13—C18	106.0 (4)	C39—C40—H40A	119.2
C14—C13—C18	122.3 (4)	C40—C41—C42	116.9 (5)
C15—C14—C13	116.8 (4)	C40—C41—H41A	121.5
C15—C14—H14A	121.6	C42—C41—H41A	121.5
C13—C14—H14A	121.6	C41—C42—N8	132.0 (5)
C14—C15—C16	121.7 (5)	C41—C42—C37	122.5 (5)
C14—C15—H15A	119.1	N8—C42—C37	105.5 (4)
C16—C15—H15A	119.1	N7—C43—N8	113.9 (4)
C15—C16—C17	121.4 (5)	N7—C43—C44	125.1 (4)
C15—C16—H16A	119.3	N8—C43—C44	121.0 (4)
C17—C16—H16A	119.3	O4—C44—C45	112.6 (4)
C16—C17—C18	117.9 (4)	O4—C44—C43	110.1 (3)
C16—C17—H17A	121.1	C45—C44—C43	111.8 (4)
C18—C17—H17A	121.1	O4—C44—H44A	107.4
C17—C18—N3	131.0 (4)	C45—C44—H44A	107.4
C17—C18—C13	119.9 (4)	C43—C44—H44A	107.4
N3—C18—C13	109.2 (4)	C44—C45—H45A	109.5
N3—C19—N4	112.8 (4)	C44—C45—H45B	109.5
N3—C19—C20	125.7 (4)	H45A—C45—H45B	109.5
N4—C19—C20	121.5 (4)	C44—C45—H45C	109.5
O2—C20—C19	105.9 (3)	H45A—C45—H45C	109.5
O2—C20—C21	111.4 (4)	H45B—C45—H45C	109.5
C19—C20—C21	112.3 (3)	N8—C46—C47	111.8 (4)
O2—C20—H20A	109.0	N8—C46—H46A	109.3
C19—C20—H20A	109.0	C47—C46—H46A	109.3
C21—C20—H20A	109.0	N8—C46—H46B	109.3
C20—C21—H21A	109.5	C47—C46—H46B	109.3
C20—C21—H21B	109.5	H46A—C46—H46B	107.9
H21A—C21—H21B	109.5	C48—C47—C46	124.4 (6)
C20—C21—H21C	109.5	C48—C47—H47A	117.8
H21A—C21—H21C	109.5	C46—C47—H47A	117.8
H21B—C21—H21C	109.5	C47—C48—H48A	120.0
N4—C22—C23	110.8 (3)	C47—C48—H48B	120.0
N4—C22—H22A	109.5	H48A—C48—H48B	120.0
C23—C22—H22A	109.5	C7—N1—C1	105.2 (4)
N4—C22—H22B	109.5	C7—N2—C6	106.7 (3)
C23—C22—H22B	109.5	C7—N2—C10	128.0 (4)
H22A—C22—H22B	108.1	C6—N2—C10	125.2 (4)
C24—C23—C22	124.2 (6)	C19—N3—C18	105.6 (3)
C24—C23—H23A	117.9	C19—N4—C13	106.4 (3)
C22—C23—H23A	117.9	C19—N4—C22	128.3 (4)
C23—C24—H24A	120.0	C13—N4—C22	125.1 (4)
C23—C24—H24B	120.0	C31—N5—C30	104.2 (4)
H24A—C24—H24B	120.0	C31—N6—C25	106.2 (3)
C26—C25—C30	123.1 (4)	C31—N6—C34	128.3 (4)
C26—C25—N6	131.7 (4)	C25—N6—C34	125.5 (4)
C30—C25—N6	105.2 (4)	C43—N7—C37	104.1 (4)
C27—C26—C25	115.7 (5)	C43—N8—C42	106.2 (3)
C27—C26—H26A	122.2	C43—N8—C46	128.0 (4)
C25—C26—H26A	122.2	C42—N8—C46	125.7 (4)
C28—C27—C26	122.4 (5)	C8—O1—H1B	104.2
C28—C27—H27A	118.8	C20—O2—H2B	98.1
C26—C27—H27A	118.8	C32—O3—H3B	109.5
C29—C28—C27	121.6 (5)	C44—O4—H4B	109.5

### Hydrogen-bond geometry (Å, º)

**Table:**

*D*—H···*A*	*D*—H	H···*A*	*D*···*A*	*D*—H···*A*
O1—H1*B*···N3^i^	0.82	1.99	2.801 (4)	167
O2—H2*B*···N1^ii^	0.82	2.05	2.811 (4)	155
O3—H3*B*···O1^iii^	0.82	1.99	2.808 (4)	175
O4—H4*B*···O2^i^	0.82	1.97	2.787 (4)	176

Symmetry codes: (i) *x*+1, *y*, *z*; (ii) *x*−1, *y*+1, *z*; (iii) *x*−1, *y*, *z*.

## References

[bb1] Garuti, L., Roberti, M. & Cermelli, C. (1999). *Bioorg. Med. Chem. Lett.* **9**, 2525–2530. 10.1016/s0960-894x(99)00429-1 10498201

[bb2] Matsuno, T., Kato, M., Sasahara, H., Watanabe, T., Inaba, M., Takahashi, M., Yaguchi, S. I., Yoshioka, K., Sakato, M. & Kawashima, S. (2000). *Chem. Pharm. Bull.* **48**, 1778–1781. 10.1248/cpb.48.1778 11086914

[bb3] Rigaku (2005). *CrystalClear* Rigaku Corporation, Tokyo, Japan.

[bb4] Sheldrick, G. M. (2008). *Acta Cryst.* A**64**, 112–122. 10.1107/S0108767307043930 18156677

[bb5] Stibrany, R. T. (2001). US Patent No. 6,180,788.

[bb6] Stibrany, R. T., Schugar, H. J. & Potenza, J. A. (2002). *Acta Cryst.* E**58**, o1142–o1144.

[bb7] Xia, R. & Xu, H.-J. (2008). *Acta Cryst.* E**64**, o1223. 10.1107/S1600536808013871 PMC2961813 21202860

